# Recurrent neural networks for generalization towards the vessel geometry in autonomous endovascular guidewire navigation in the aortic arch

**DOI:** 10.1007/s11548-023-02938-7

**Published:** 2023-05-28

**Authors:** Lennart Karstensen, Jacqueline Ritter, Johannes Hatzl, Floris Ernst, Jens Langejürgen, Christian Uhl, Franziska Mathis-Ullrich

**Affiliations:** 1 Fraunhofer IPA, Theodor-Kutzer-Ufer 1-3, 68167 Mannheim, Germany; 2 Department of Vascular and Endovascular Surgery, University Hospital Heidelberg, Im Neuenheimer Feld 420, 69120 Heidelberg, Germany; 3 Institute for Robotics and Cognitive Systems, University of Lübeck, 23562 Lübeck, Germany; 4 Department Artificial Intelligence in Biomedical Engineering (AIBE), Friedrich-Alexander University Erlangen-Nürnberg, Werner-von-Siemens-Straße 61, 91052 Erlangen, Germany

**Keywords:** Endovascular intervention, Recurrent neural network, Deep reinforcement learning, Autonomous, Generalization

## Abstract

**Purpose:**

Endovascular intervention is the state-of-the-art treatment for common cardiovascular diseases, such as heart attack and stroke. Automation of the procedure may improve the working conditions of physicians and provide high-quality care to patients in remote areas, posing a major impact on overall treatment quality. However, this requires the adaption to individual patient anatomies, which currently poses an unsolved challenge.

**Methods:**

This work investigates an endovascular guidewire controller architecture based on recurrent neural networks. The controller is evaluated in-silico on its ability to adapt to new vessel geometries when navigating through the aortic arch. The controller’s generalization capabilities are examined by reducing the number of variations seen during training. For this purpose, an endovascular simulation environment is introduced, which allows guidewire navigation in a parametrizable aortic arch.

**Results:**

The recurrent controller achieves a higher navigation success rate of 75.0% after 29,200 interventions compared to 71.6% after 156,800 interventions for a feedforward controller. Furthermore, the recurrent controller generalizes to previously unseen aortic arches and is robust towards size changes of the aortic arch. Being trained on 2048 aortic arch geometries gives the same results as being trained with full variation when evaluated on 1000 different geometries. For interpolation a gap of 30% of the scaling range and for extrapolation additional 10% of the scaling range can be navigated successfully.

**Conclusion:**

Adaption to new vessel geometries is essential in the navigation of endovascular instruments. Therefore, the intrinsic generalization to new vessel geometries poses an essential step towards autonomous endovascular robotics.

**Supplementary Information:**

The online version contains supplementary material available at 10.1007/s11548-023-02938-7.

## Introduction

Endovascular interventions are the state-of-the-art treatment for many vascular diseases, which include the common diseases of heart attack and stroke. For treatment, a guidewire and catheter are inserted into the patient’s vessel system and navigated to the lesion, e.g. the coronary arteries for a heart attack or cerebral arteries for a stroke. At the lesion, treatment is performed through the catheter, which may include placing a stent, or mechanically removing the stenosis.

To navigate to the lesion, the instruments are manipulated by translation, i.e. insertion and retraction, and rotation, i.e. twisting, of the instruments from outside the patient’s body. Feedback of the current position of the instrument’s tip is obtained through continuous fluoroscopy imaging, a low dose variant of X-ray imaging. In a normal fluoroscopy image, the guidewire and catheter are visible, but arteries and veins cannot be differentiated from the surrounding tissue. By injecting contrast agent, which makes the vascular system radiopaque, vessels can be made visible fluoroscopy image. By creating a snapshot with contrast agent, a roadmap can be overlayed with the fluoroscopy image showing the vessel geometry. Too much contrast agent can pose a health risk and creating and updating the roadmap takes time, therefore these methods are only applied when necessary.

A typical method for selective catheterization is leading with the guidewire. The guidewire’s angled tip allows probing the desired artery at bifurcations by skillful rotation and insertion such that the tip enters the desired artery. In cases where the guidewire alone is not able to probe the desired artery, it is necessary to utilize guidewire and catheter concurrently. The shaped tips of both instruments influence each other and can be modified by skillfully changing overlap and rotation allowing to probe difficult vessels. When the guidewire sits firmly in the artery, the endovascular surgeon pushes the more flexible catheter over it [1, 2].

During such a procedure, the surgeon must continuously stand in close proximity to the patient at the operating table and is exposed to radiation. Best practices can already significantly reduce radiation exposure, but a further reduction is still a reasonable goal for the health of the surgeon [3, 4]. Automation of the endovascular navigation will allow the physician to stay out of the radiation zone during the navigation.

Remote and rural areas have a significantly reduced access to endovascular treatments compared to urban areas [5]. One approach to solve this problem is telesurgery, which is shown as feasible in research settings [6]. A comprehensive application of telesurgery is not yet possible due to technical constraints, e.g. connection stability and latency requirements, and legal issues, e.g. liability or validation of the telesurgery networks [7]. Autonomous navigation can supplement telesurgery by lessening the requirements on the communication network or can be utilized where no telesurgery network or skilled physician is available.

Additionally, cognitive and physical strain of the operating surgeon can be reduced by an autonomous system such that physicians can change their attention focus from the visuo-motoric task of manipulating the endovascular instruments to high-level intervention decisions.

Recent research regarding autonomous control of endovascular instruments during navigation in interventions utilize learning-based approaches [8–15]. They utilize supervised learning [8], deep-q-networks [9, 10], asynchronous advantage actor-critic [11], deep deterministic policy gradients with hindsight experience replay [12], generative adversarial imitation learning and proximal policy optimization [13], soft actor critic [14] or discrete soft actor critic with auto-encoder [15]. These approaches are all trained on one vessel geometry and show reduced success [13] on a different geometry.

Learning-based controllers utilizing neural networks show a high potential of autonomously navigating endovascular instruments. However, the lacking generalization to new geometries is problematic, as each patient has a unique vascular system. Generalization to previously unseen environments is a major challenge in reinforcement learning research and is an essential component to deploying algorithms in real world scenarios [16].

This work builds on the results of [12], where testbench performance decreased compared to the simulation results, partly due to the soft vessel geometry changing shape in the testbench. Here, we design a learning-based controller utilizing recurrent neural networks to autonomously navigate varying geometries. Navigation is performed in synthetic three-dimensional aortic arches to targets in the supraaortal arteries. Probing the supraaortal arteries is an important step in the treatment of cerebral arteries. Strong anatomical variations occur rarely and are not considered. The intervention is performed with a guidewire only and without geometry information. The utilization of contrast agent to gain geometry information and utilization of a catheter are not regarded. Instrument characteristics remain constant. Subsequently, we examine the generalizability of the controller by reducing the training context, such that evaluation is performed in aortic arches, which were not encountered during training. To perform the in-silico experiments we introduce a parametrizable endovascular environment with aortic arches randomized by arch type, specific geometry, width scaling and height scaling.

## Methods and materials

### Navigation task

**Fig. 1:**
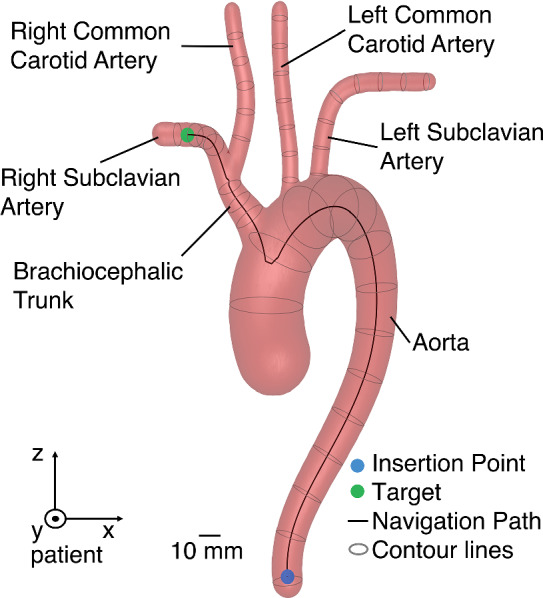
Aortic arch model with the artery nomenclature, insertion point, a possible target, guidewire's path to the target and the patient coordinate system

Here, we consider the navigation through the aortic arch from the descending aorta to the supraaortal arteries. An example of one aortic arch model, the insertion point, a possible target point, the path to the target and the artery nomenclature are presented in Fig. 1. The controller's task is to navigate a guidewire from the insertion point at the caudal end of the models to a target randomly sampled from the centerlines of the supraaortal arteries. For each navigation attempt the vessel geometry is randomized. Feedback during the navigation is given as two-dimensional (*x*′, *z*′) tracking coordinates of the guidewire. No feedback about the vessel geometry is given. The tracking coordinate system is a rotated projection of the patient coordinate system to a left anterior oblique (LAO) projection with a − 25° rotation around the *z*-axis and 5° around the *x*-axis. This projection is normally utilized when navigating the aortic arch, as the visibility of the aortic arch is maximized in the fluoroscopy images. Therefore, the feedback is equivalent to the two-dimensional tracking that would be extracted from such fluoroscopy images.

Preliminary experiments with manually performed interventions with full visibility of the vessel geometry and unlimited duration showed that 76.3% of the random geometry and target combinations can be reached within a maximum of 19 s. Training of the controller is performed on random geometry/target combinations and evaluation on 1000 preselected reachable geometry/target combinations. The success criterion is whether the target is reached within 60 s, allowing the controller to make failures and correct them.

For each navigation attempt, a new aortic arch is generated by a parametrizable aortic arch generator. Four parameters are considered: arch type, specific geometry of each artery in the arch type, height scaling and width scaling. Height and width correspond to the patient’s *z*-axis and *xy*-axes, respectively.

**Fig. 2:**
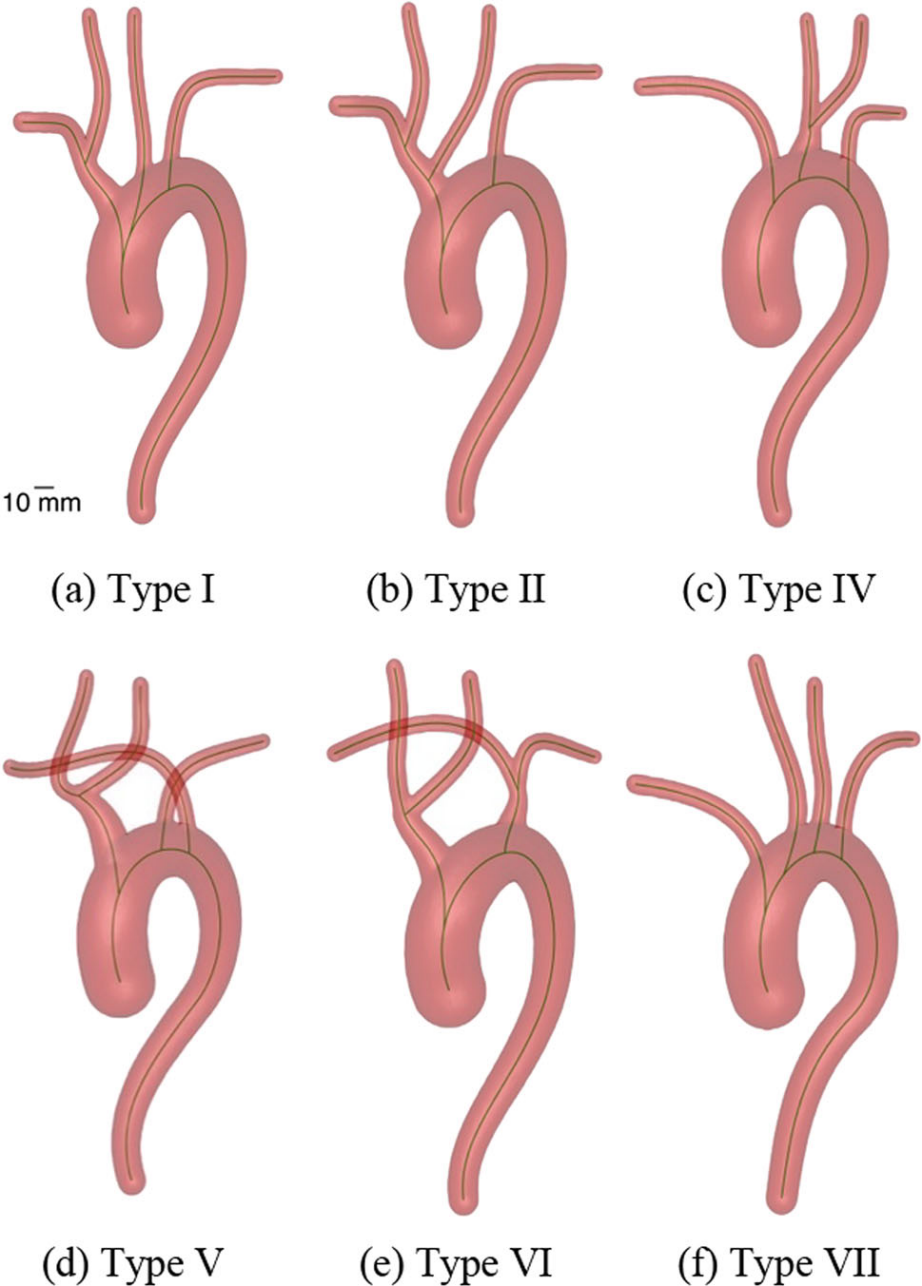
Aortic arch type variations available in the aortic arch generator. The centerlines of the arteries are shown in green

Aortic arch types are derived from Natsis et al. [17]. Here, we utilize types I, II, IV, V, VI and VII as shown in Fig. 2a–f. Types III and VIII are neglected as in these types the left vertebral artery and the thyroidea ima artery emerge directly from the aortic arch, two arteries, which are not considered in our study.

**Fig. 3:**
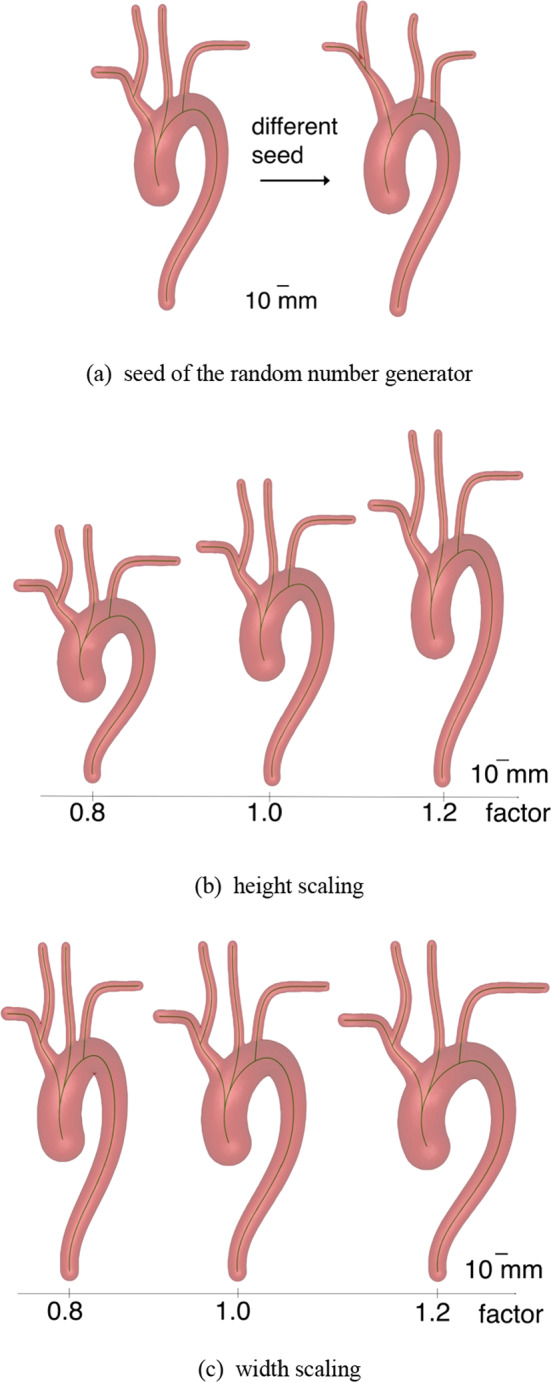
Aortic arch type variations for height and width scaling and the seed for the detailed geometry. The centerlines of the arteries are shown in green

After selecting an arch type, the specific geometry of the arteries is calculated utilizing cubic hermite splines with randomized spline points. Each artery is defined by a finite number of spline points, e.g. the aorta and the left subclavian artery are defined by four and two points, respectively. Each spline point is defined by its coordinates, the artery diameter and the parametric derivative of the coordinates and the artery diameter with respect to the independent spline variable. Coordinates and artery diameters, as well as their parametric derivatives are derived from normal distributions. Starting points of arteries are constrained to the centerline of the preceding artery. A specific geometry can be reproduced by setting a certain seed to the random number generator for the spline points. In Fig. 3a two different seeds for a type I aortic arch are shown.

In addition, the aortic arch can be independently scaled in its height and width, while the diameter of the arteries remains constant. The effect of scaling the height is depicted in Fig. 3b and scaling the width in Fig. 3c.

The navigation task is modelled in a finite-element-simulation based on the SOFA framework [18] with the BeamAdapter plugin [19]. The guidewire is modelled as a one-dimensional finite-element-model based on Kirchhoff’s rod theory confined by the vessel-phantom walls. Phantom walls are assumed rigid and the lumen empty. Friction between wall and guidewire as well as guidewire stiffness have been adapted from [12] where they were iteratively tuned to mimic real guidewire behaviour. The simulation receives guidewire rotation and translation speed as input. Rotation and translation are applied at the proximal end of the guidewire. The output is the guidewire position as coordinate points, directly extracted from the finite-element-model of the simulation. Rotational speed is limited to 180°/s and translational speed to 40 mm/s.

### Controller architecture and training procedure

**Fig. 4:**
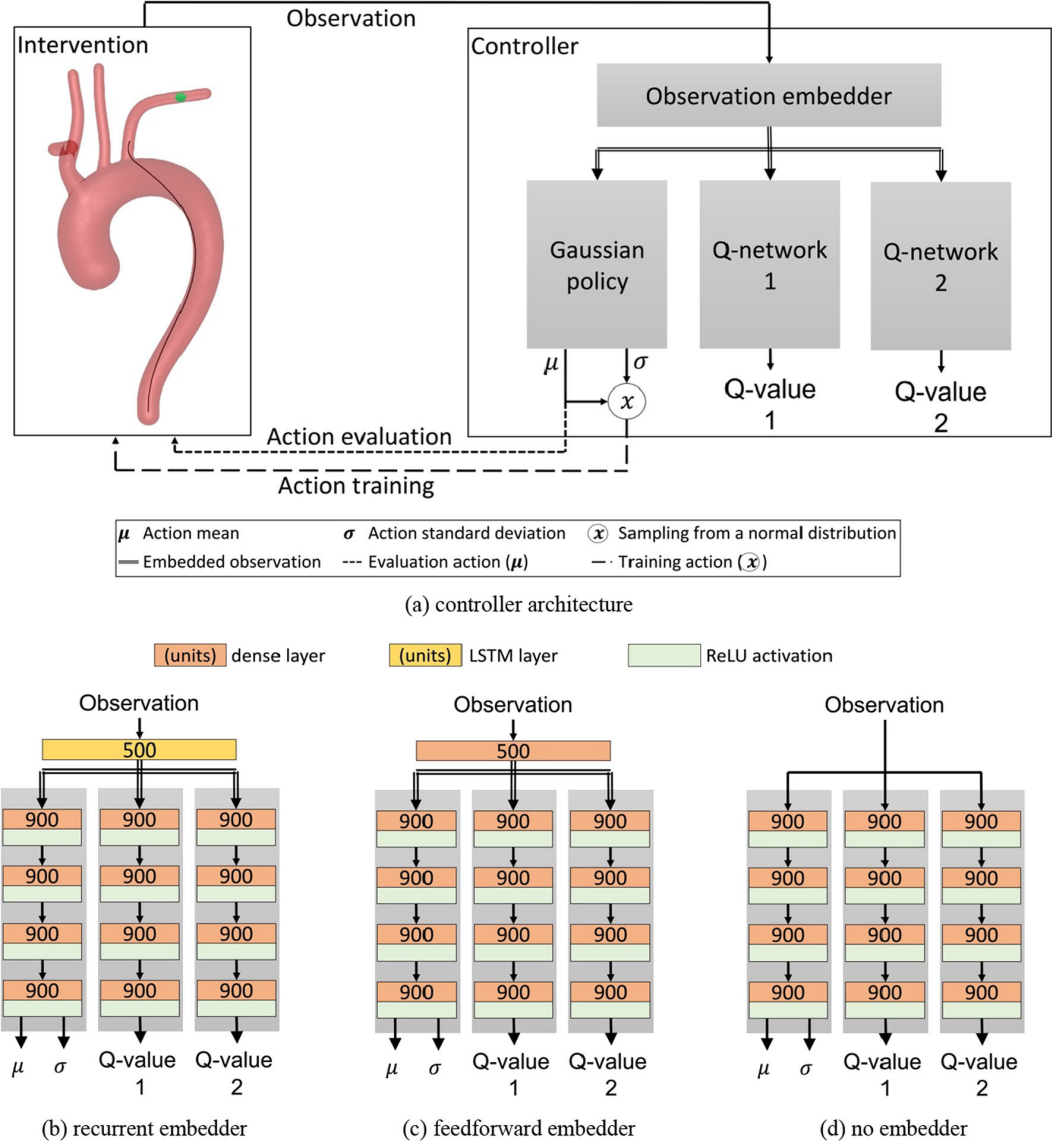
**a** Architecture of the proposed controller based on a soft actor-critic design. Reward and action input for the Q-networks are neglected. **b–d** Neural-network architectures of the controllers with recurrent-embedder, feedforward embedder and no embedder, respectively

To autonomously navigate the guidewire in arbitrarily generated aortic arches, we introduce a neural-network-based controller, which incorporates a recurrent layer trained by the reinforcement learning method soft actor critic [20]. The recurrent architecture adds a long short-term memory (LSTM) layer to the strictly feedforward architecture utilized in the state of the art. The LSTM layer serves as an observation embedder, which learns a useful trajectory dependent state representation, while the following feedforward layers learn the control of the guidewire. The recurrent layer is expected to improve performance in this partially observable navigation task (the vessel geometry is not observable), as shown for other partially observable environments [21]. Assumably, the LSTM remembers information about the geometry of the vessel system as proposed by Ma et al*.* [22] for robotic indoor navigation. The observation embedder is updated with the q1-network only. Controllers with feedforward embedder and without embedder serve as baseline. The controller and network architectures are shown in Fig. 4.

The controller receives an observation as input and the gaussian policy network provides a mean $\mu$ and standard deviation $\sigma$ of a normal distribution for the next action. For training, an action is sampled from the normal distribution. For evaluation, mean $\mu$ is taken as action directly, thus the behaviour becomes deterministic. Network architectures have been chosen based on preliminary experiments with the recurrent-based controller where learning rate, embedder and network size were randomly sampled. The parameters with the best success rate have been chosen.

The action, i.e. the output of the gaussian policy, is defined as the guidewire’s rotation and translation speed. The observation is defined as current and last guidewire position, target position and action leading from the last to the current position. Position data are given as (*x*′,* z*′)*-*coordinates in the tracking coordinate system, relative to the insertion point. The guidewire position is provided as three points on the guidewire tip, described as (*x*′*, z*′)_*i*=1,2,3_. The points are spaced evenly and 2 mm apart from each other, and (*x*′*, z*′)_1_ is coincident with the guidewire tip. The target position is given as the (*x*′*, z*′)*-*coordinates of the current target. A video description of the observation can be found in Online Resource 1.

For training the controller, navigation tasks are performed for 2 × 10^7^ exploration steps. An exploration step is defined as performing one cycle of the control loop in the exploration phase. One navigation task, i.e. navigating the guidewire from the start to a target, is defined as an episode. An episode is considered complete when the target is reached within a threshold of 5 mm. During training a timeout is introduced for computational efficiency after 150 exploration steps (i.e. 20 s) without reaching the target. The control frequency is 7.5 Hz, representing a fluoroscopy image frequency of 7.5 images/s. The geometry is changed every episode.

The reward per step, *R*, is defined as:

$$ R =  - 0.005 - 0.001 \cdot \Delta {\text{ pathlength}} + \begin{array}{*{20}c}    {\left\{ {\begin{array}{*{20}l}    {1.0}  \\    0  \\   \end{array} } \right.} & {\begin{array}{*{20}l}    {{\text{if~}}\,{\text{target~}}\,{\text{reached}}}  \\    {{\text{else}}}  \\   \end{array} }  \\   \end{array} $$

The pathlength is defined as the distance of the guidewire tip to the target along the centerlines of the arteries and Δpathlength = pathlength_t=0_ − pathlength_t=−1_. For each aortic arch the pathlength is calculated based on the centerlines of the individual geometry. This is an adaption of the reward utilized in [12] enhanced by the dense feature from [14] and [15]. Parameters for the constant penalty and change in pathlength are chosen such that they approximately equalize each other with an optimal action. Preliminary experiments showed good results. Optimization of the reward might have an impact on the training efficiency and success rate but is not considered in this study.

Every 2.5 × 10^5^ exploration steps, the performance of the controller is evaluated for 1000 episodes. Performance is defined as the percentage of evaluation episodes where the controller can successfully reach the target.

### Evaluation

For the initial evaluation the training and evaluation environment are maintained identical. All six aortic arch types are used, the random number generator to determine the specific geometry is initialized randomly, and scaling is performed over the full range of 0.7–1.3. The proposed controller with recurrent embedder is compared to the controllers with feedforward embedder and no embedder. All controllers are trained with the same set of hyperparameters. Additionally, the controller with recurrent embedder is evaluated on an aortic arch model of a 23-year-old healthy male patient. The vascular model is obtained from the Vascular Model Repository (#94) [23].

To test generalization capabilities, the variety of aortic arch geometries utilized for training is reduced. We examine the capability of the controllers with recurrent and feedforward embedder to generalize towards specific geometry and the scaling of the aortic arches regarding interpolation and extrapolation.

To examine generalization towards the specific geometry, the random aortic arch generator is limited to type I aortic arches with a scale factor of 1.0, while the seed of the random number generator is varied. A finite set of random number generator seeds is introduced during exploration, while evaluation seeds were sampled from a different set. We train controllers with 1, 2, 4, 8, 16, 32, 64, 128, 256, 512, 1024, 4096, 8192, 16,384 and 32,768 seeds and evaluate on 231 reachable geometry/target combinations. A controller trained on seeds randomly sampled between 0 to 2^31^ for every episode serves as baseline.

For scaling generalization, the random aortic arch generator is limited to type I aortic arches with the single seed 13 for the random number generator, while the scaling range is varied. Interpolation and extrapolation are examined.

For interpolation, the scaling range utilized during training is evenly distributed at the upper and lower end of the full scaling range of 0.7–1.3, e.g. a controller trained with 60% scaling range will utilize the ranges 0.7–0.88 and 1.12–1.3 during training and 0.7–1.3 during evaluation. We train controllers on 10–100% of the full scaling range in 10% intervals.

For extrapolation, the scaling range utilized during training is evenly distributed in the middle of the full scaling range of 0.7–1.3, e.g. a controller trained with 60% scaling range will utilize the range 0.82–1.18 during training and 0.7–1.3 during evaluation. A controller trained on the full scaling range serves as baseline. We train controllers on 10–100% of the full scaling range in 10% intervals.

## Results

**Fig. 5:**
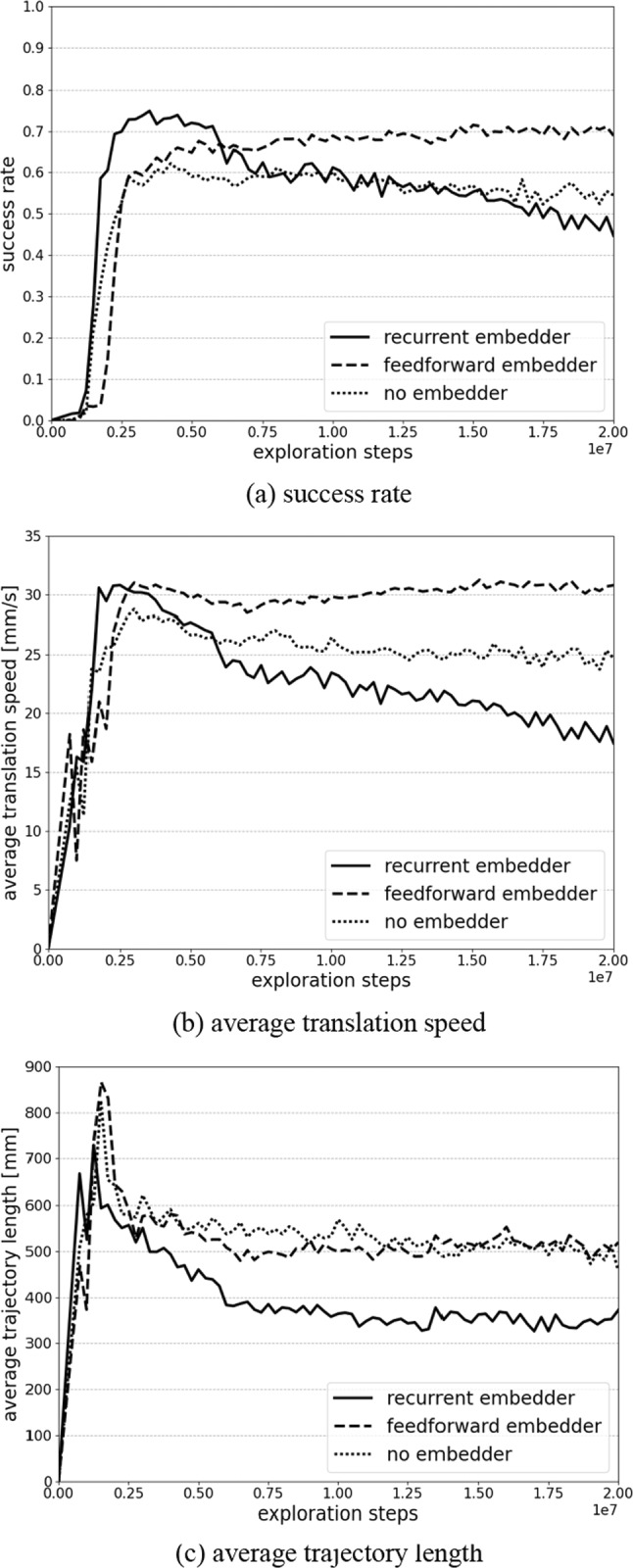
**a** Success rates, **b** average translation speed and **c** average trajectory length during training for the recurrent controller and the feedforward controller with the full aortic arch distribution during training

Figure 5 depicts the success rate, average translation speed and average trajectory length during training of the initial evaluation. The recurrent embedder reaches a success rate of 75.0%, average translation speed of 30.1 mm/s and average trajectory length of 499.1 mm after 3.5 × 10^6^ exploration steps (29,200 episodes). The feedforward embedder reaches its maximum of 71.5% success with 30.8 mm/s and 507.8 mm after 15.0 × 10^6^ exploration steps (156,800 episodes). The controller without embedder reaches 62.2% success with 28.0 mm/s and 560.7 mm after 4.25 × 10^6^ exploration steps (33,200 episodes).

**Fig. 6:**
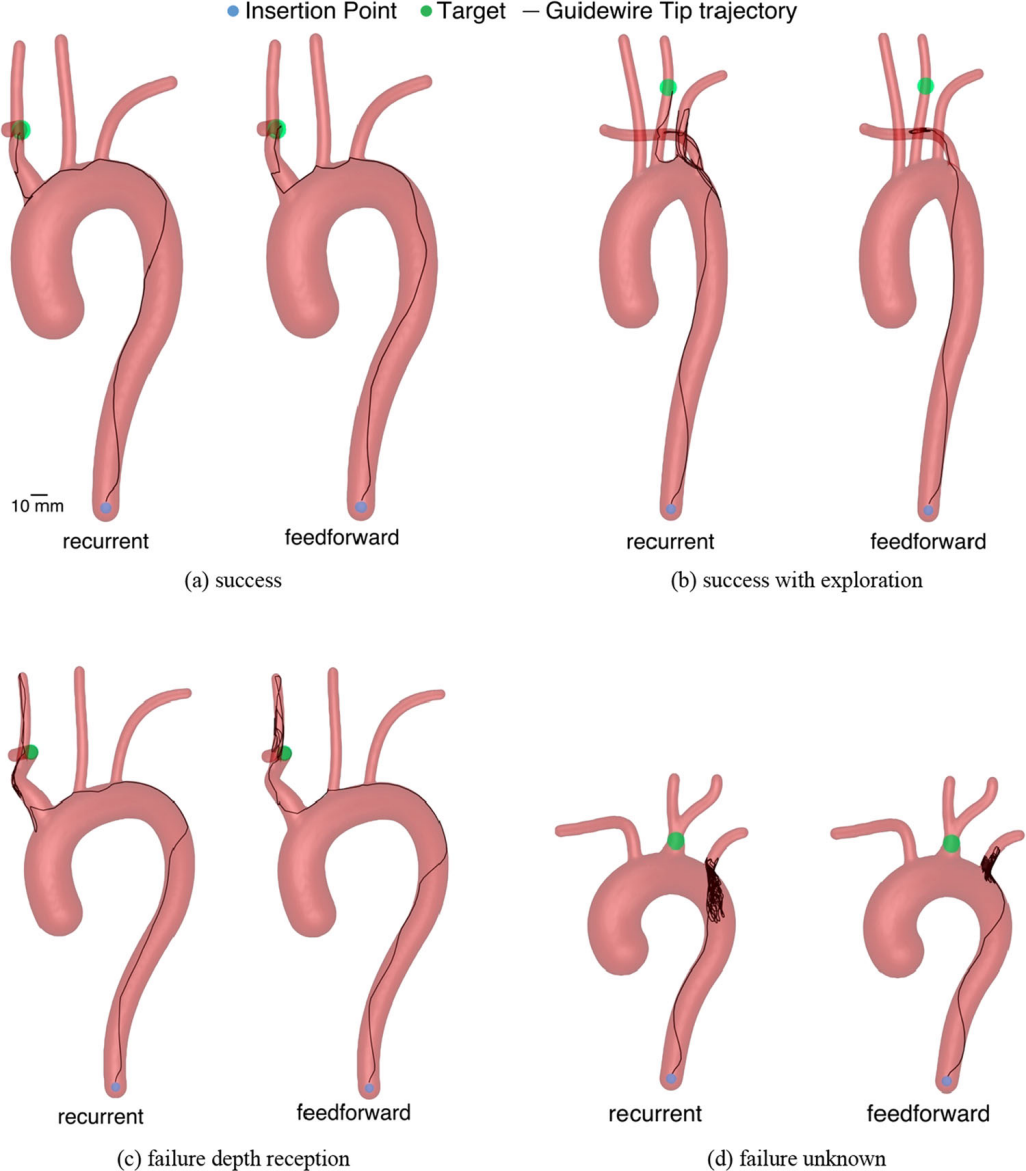
Example trajectories of controller with recurrent and feedforward embedder with **a** success, **b** success if arteries are explored, **c** failure due to a lack of depth reception and **d** unknown failure

Figure 6 shows trajectories of the guidewire tip of four exemplary navigation tasks for the controllers with recurrent and feedforward embedder. In Fig. 6a both reach the target directly, in 6b only the recurrent controller reaches the target in the left common carotid artery after exploring the right and left subclavian arteries and the feedforward controller enters the right subclavian artery and remains there, in 6c both controllers navigate to the correct coordinates in the two-dimensional tracking coordinate system but cannot reach the target which is in the right subclavian artery behind the guidewire tip and in 6d both controllers fail without apparent reason. Videos of the navigation process can be found in Online Resource 1.

**Fig. 7:**
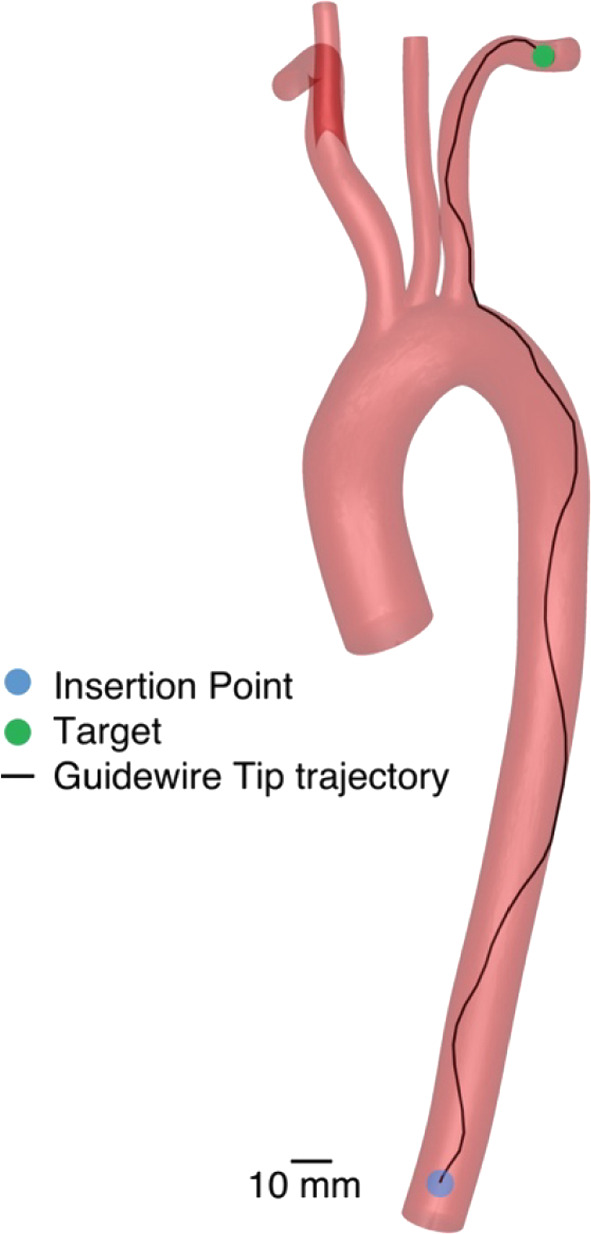
Guidewire tip trajectory for the recurrent controller in an aortic arch model from a real patient

On the vessel geometry of the real patient, the recurrent controller achieves a success rate of 29%. The success rate strongly varies between the target branches with 78.6% in the left subclavian artery, 31.8% in the left common carotid artery and 0% in the brachiocephalic trunk, right subclavian and right common carotid artery. An example for a successful trajectory can be seen in Fig. 7 and Online Resource 1.

**Fig. 8:**
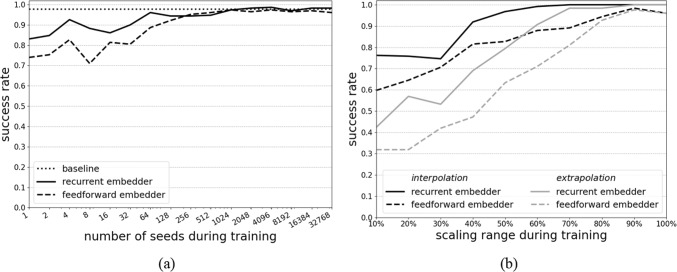
Success rates for generalization experiments for the variation of **a** number of seeds and **b** scaling range percentage

The experimental evaluation of the controllers’ generalization behaviour is shown in Fig. 8a and b. For seed variation the baseline reaches a success rate of 97.8% after 29,100 episodes. Performance increases with the number of seeds utilized during training. The controller with recurrent embedder reaches the baseline success rate trained with 2048 seeds. The controller with feedforward embedder reaches a maximum of 97.4% trained with 1024 seeds.

Results regarding generalization towards scaling the aortic arch are shown in Fig. 8b. Performance increases with the size of the scaling range utilized during training. For the recurrent embedder, interpolation starts at 76.2% success rate and reaches 100% with 70% scaling range and extrapolation starts at 42.3% and reaches 100% success rate with 90% scaling range. For the feedforward embedder, interpolation starts at 59.7% success rate and reaches 98.4% with 90% scaling range and extrapolation starts at 31.9% success rate and reaches 97.6% with 90% scaling range.

It is noticeable that the success rate is not steadily improving with increasing similarity between the training and evaluation distribution but fluctuates by up to 2.4% generally and 6.5% if training and evaluation environment differ strongly.

One training with 2 × 10^7^ exploration steps took 29.1 h on a computer equipped with an AMD Ryzen Threadripper 3970x CPU and Nvidia RTX3090 GPU and required 68 GB RAM.

## Discussion

### Recurrent architecture for patient adaptability

The recurrent architecture has a slight advantage in patient adaptability to the feedforward architecture improving success rate from 71.5 to 75.0%. Analysis of the guidewire tip trajectories from Fig. 6b and Online Resource 1 suggest, that the recurrent architecture can probe for the correct vessel when the target branch is not unambiguously from the target coordinates.

The reduction of required episodes from 156,800 to 29,200 is an important advantage of the recurrent architecture as it is costly to obtain intervention data in real interventions.

### Generalization to unknown aortic arch geometries

Both controllers show generalization capabilities regarding the number of specific geometries and scaling ranges utilized during training. In all cases, the recurrent embedder supersedes the feedforward embedder.

As expected, the performance improves with increasing amount of training context but doesn’t improve steadily. It fluctuates with increasing training context and sometimes even supersedes the baseline. Presumably, the initialization state of the neural networks is important for the resulting performance.

Generalization is achieved with 2048 specific geometries, with 90% of the scaling range for extrapolation, and 70% for interpolation. When training on patient data, edge cases should be included, as extrapolation capabilities are low, but cases in the middle of the distribution may be missing because of the comparatively good interpolation capabilities.

### Limitations

The recurrent controller shows generalization capabilities to unseen aortic arch geometries but navigation success for any other part of the vessel system is not guaranteed. However, during an endovascular intervention several vessel structures must be navigated to reach a typical stroke lesion. Thus, the presented architecture requires a distinct controller for each vessel structure. Additionally, we consider only physiological aortic arches and ignore pathological aortic arches. In theory this methodology can be applied to other organs where the underlying structure is similar among patients, e.g. femoral arteries, by adjusting the training environment, but cannot be applied to organs where the vessel structure differs randomly, e.g. prostate or mesenteric arteries.

The controller is trained on synthetic aortic arches. Evaluation on the model of a real aortic arch shows mixed results, depending on the target branch. For adaption to real patients either the aortic arch generator should be improved, or real aortic arch models need to be used during training.

Transfer from simulation to clinical application poses further challenges caused by the so-called sim-to-real gap. Inaccuracies and delays of the tracking algorithm and variations in the dynamics of the guidewire and interaction between guidewire and vessel system may occur.

A further limitation of our approach is the selected performance metric that purely considers whether the guidewire tip successfully reaches the target. However, in endovascular interventions a gentle navigation approach is required to avoid injuring the patient.

## Conclusion

Adaptability to new vessel geometries is an essential requirement for a controller that is to navigate endovascular guidewires autonomously. For this purpose, we present a controller architecture based on recurrent neural networks trained with reinforcement learning. The recurrent controller shows an increase in performance, sample efficiency and generalization capabilities compared to a purely feedforward controller.

Due to patient individuality, an autonomous guidewire controller is required to adapt to each unique vessel geometry. Extracting patient-specific characteristics prior to the intervention is often not feasible. Therefore, the intrinsic generalization to new vessel geometries poses an essential step towards autonomous endovascular robotics.

## Supplementary Information

Below is the link to the electronic supplementary material.

Supplementary file1 (MP4 55056 kb)

## Declarations

### Supplementary material

The online version contains supplementary material. The source code of the aortic arch generator can be found on https://github.com/lkarstensen/2023_IJCARS_aortic_arch_generator.
